# [^18^F]AlF-NOTA-pentixather PET/CT of CXCR4 in patients with suspected primary hyperaldosteronism

**DOI:** 10.7150/thno.100848

**Published:** 2024-10-28

**Authors:** Limeng He, Yan Yang, Xu Cao, Xianjun Zhu, Nan Liu, Xiaoyuan Chen, Jingjing Zhang, Wei Zhang

**Affiliations:** 1 Department of Nuclear Medicine, Sichuan Provincial People's Hospital, School of Medicine, University of Electronic Science and Technology of China, Chengdu 610072, China.; 2 Department of Endocrinology & Metabolism, Sichuan Provincial People's Hospital, University of Electronic Science and Technology of China, Chengdu 610072, China.; 3 Department of Diagnostic Radiology, Yong Loo Lin School of Medicine, National University of Singapore, Singapore 119074, Singapore.; 4 Clinical Imaging Research Centre, Centre for Translational Medicine, Yong Loo Lin School of Medicine, National University of Singapore, Singapore 117599, Singapore.; 5 Theranostics Center of Excellence, Yong Loo Lin School of Medicine, National University of Singapore, 11 Biopolis Way, Helios, Singapore 138667, Singapore.; 6 Nanomedicine Translational Research Program, NUS Center for Nanomedicine, Yong Loo Lin School of Medicine, National University of Singapore, Singapore 117597, Singapore.; 7 Departments of Surgery, Chemical and Biomolecular Engineering, and Biomedical Engineering, Yong Loo Lin School of Medicine and College of Design and Engineering, National University of Singapore, Singapore 119074, Singapore.; 8 Institute of Molecular and Cell Biology, Agency for Science, Technology, and Research (A*STAR), 61 Biopolis Drive, Proteos, Singapore 138673, Singapore.

**Keywords:** [^18^F]AlF-NOTA-pentixather, [^18^F]AlF-NOTA-T140, PET/CT, chemokine receptor, Primary aldosteronism, Aldosterone-producing adenoma

## Abstract

**Background:** Distinguishing unilateral aldosterone-producing adenomas (APA) from idiopathic hyperaldosteronism (IHA), nonfunctional adrenal adenoma (NFA), and pheochromocytoma (PHEO) within primary aldosteronism (PA) presents a significant challenge. Studies have demonstrated high levels of chemokine receptor (CXCR) 4 expression in APA, thereby validating the use of ^68^Ga-labeled CXCR4 PET/CT for detecting APA. This study evaluates the efficacy of [^18^F]AlF-NOTA-pentixather PET/CT in distinguishing APA from other PA types.

**Methods:** In the initial experiment, a comparative analysis was conducted to evaluate the diagnostic efficacy of [^18^F]AlF-NOTA-T140 PET/CT and [^18^F]AlF-NOTA-pentixather PET/CT for APA in 3 patients with PA. Based on the preliminary findings, [^18^F]AlF-NOTA-pentixather PET/CT was subsequently performed on 45 patients with suspected PA and 5 controls. Lesions exhibiting higher tracer uptake than normal adrenal glands were considered positive and referred for adrenalectomy. Prior to surgery, adrenal venous sampling (AVS) was performed in 71.1% of patients to assess laterality. Postoperative follow-up was conducted in 91.1% of patients. The semi-quantitative analysis involved assessing maximum standardized uptake value (SUVmax), LLR (lesion-to-liver ratio), and lesion-to-contralateral ratio (LCR). Correlations were made between PET/CT findings, histopathology results, outcomes, and AVS.

**Results:** In terms of diagnosing APA, [^18^F]AlF-NOTA-pentixather PET/CT demonstrated a sensitivity of 100%, specificity of 91.7%, and accuracy of 95.8%. The mean SUVmax for APAs (25.62 ± 12.71, n = 24) was significantly higher compared to non-APA cases (7.24 ± 3.27, n = 24, *P* < 0.0001). An optimal SUVmax threshold of 11.60 accurately predicted the presence of APA with a sensitivity of 95.8%, specificity of 96.0%, and accuracy of 93.9%. A cutoff value for LCR at 1.38 provided 95.8% sensitivity and 92.0% specificity, while an LLR cutoff at 5.28 yielded a sensitivity rate of 91.7% and a specificity rate of 92.0%. Positive findings on PET/CT scans were completely consistent with AVS results. All patients with positive lesions derived significant benefits from surgical intervention.

**Conclusion:** [^18^F]AlF-NOTA-pentixather PET/CT seems to be highly related to AVS and could be a noninvasive method for diagnosing APA in patients with PA.

## Introduction

Primary aldosteronism (PA) is characterized by excessive aldosterone production by the adrenal glands, leading to hypertension and hypokalemia. Left untreated, it elevates the risk of cardiovascular events and renal impairment 1, 2. The primary causes of PA include idiopathic hyperaldosteronism (IHA), which is caused by unilateral or bilateral adrenal hyperplasia, and aldosterone-producing adenoma (APA), with rare cases attributed to familial hyperaldosteronism, adrenocortical carcinomas, and ectopic aldosterone-producing tumors 3, 4. Distinguishing APA from IHA, nonfunctional adrenal adenoma (NFA) as well as determining unilateral versus bilateral disease, is crucial for treatment planning. While adrenal CT imaging aids in excluding carcinomas and suggests PA subtype, its accuracy is limited for lesions <1 cm and in distinguishing APA from NFA 5, 6. Adrenal venous sampling (AVS) is considered the standard test to determine PA laterality but has variable success rates (29-74%) and risks complications. Moreover, it is invasive 7.

Nuclear medicine imaging combines the advantages of anatomical and functional imaging for diagnosing APA. Metomidate (MTO) is a selective inhibitor of 11-β-hydroxylase (CYP11B1) and aldosterone synthase (CYP11B2). ^11^C-MTO PET is used to distinguish adrenocortical tumors from non-adrenocortical tumors 8. The ability of ^11^C-MTO PET to diagnose APA is controversial, and the short half-life of ^11^C and the requirement for an on-site cyclotron may limit its clinical use 9. Chemokine receptor (CXCR) 4 is specific for stromal cell-derived factor-1 (SDF-1), with high expression in APA, significantly correlating with CYP11B2 expression 10, 11. Studies have shown that the gallium-68-labeled chemokine imaging agent ^68^Ga-pentixafor may effectively identify APA in patients with PA, potentially making ^68^Ga-pentixafor PET/CT a valuable tool in surgical decision-making for PA patients 12, 13.

Fluorine-18 offers longer imaging windows, higher spatial resolution, and wider availability of radiotracers compared to Gallium-68, making it more versatile and broadly applicable in clinical practice 14. Poschenrieder *et al.* 15 developed a fluorine-tagged CXCR4 imaging agent [^18^F]AlF-NOTA-pentixather, which showed higher CXCR4 affinity than ^68^Ga-pentixafor. Our team developed another fluorine-tagged CXCR4 imaging agent [^18^F]AlF-NOTA-T140 16, and we obtained similar results to Poschenrieder's.

Since adrenal pheochromocytoma (PHEO) and myelolipoma are difficult to differentiate from APA or NFA using conventional methods, our study has incorporated these two patient groups for statistical analysis. In the following text, we will refer to NFA, PHEO and myelolipoma collectively as non-secreting aldosterone tumors (NAT). This study aimed to prospectively evaluate the utility of [^18^F]AlF-NOTA-pentixather PET/CT in patients with suspected PA, particularly for distinguishing APA from IHA and NAT.

## Materials and methods

### Study population

We conducted a prospective study on participants with clinical suspicion of PA at our institution. Inclusion criteria were (1) severe hypertension (systolic blood pressure >150 mmHg or diastolic blood pressure >100 mmHg) or drug-resistant hypertension (blood pressure >140/90 mmHg despite combined use of three types of antihypertensive medications) with or without hypokalemia, or (2) persistent hypertension with blood pressure > 160/100 mmHg or refractory hypertension with an aldosterone-to-renin ratio (ARR) ≥ 30 (ng/dl)/(ng/ml/h) based on clinical guidelines from the subcommittee of the Endocrine Society 17, or (3) uncontrollable hypertension with unilateral or bilateral adrenal nodule(s) with clear border shown on CT, or (4) AVS results suggesting lateral aldosterone secretion. A positive saline test (aldosterone > 10 ng/dL after saline administration) or a positive captopril test (a decrease in plasma aldosterone levels by ≤ 30% after captopril administration) was regarded as a sufficient but not obligatory condition. In addition, patients who had unilateral or bilateral adrenal nodule(s) with clear borders shown on CT but without hypertension and ARR < 30 (ng/dl)/(ng/ml/h) were included in the NAT group. Exclusion criteria were (1) pregnant women and breastfeeding mothers; (2) individuals who are unable to remain calm and still in a supine position for twenty minutes due to conditions such as claustrophobia or severe illness. The patients who underwent adrenalectomy or had follow-up data were included in our final analysis. The study was approved by the Institutional Review Board (IRB) of the Sichuan Provincial People's Hospital and listed at clinicaltrials.gov (ID: NCT05815069). Written informed consent was obtained from all participants prior to [^18^F]AlF-NOTA-T140 PET/CT and ^18^F-AlF-NOTA-pentixather PET/CT.

### AVS

AVS without adrenocorticotropic hormone (ACTH) stimulation was performed between 08:00 and 12:00 AM. The selectivity index (SI) was defined as the [cortisol (adrenal vein) / cortisol (peripheral vein)], with successful catheterization of the adrenal vein defined as SI ≥ 2. The lateralization index (LI) was defined as [aldosterone-to-cortisol ratio (ACR)] / [ACR of non-dominant adrenal vein], and contralateral suppression is defined as [ACR of non-dominant adrenal vein] < [ACR of peripheral vein]. A diagnosis of APA was made for patients with LI ≥ 4 or LI 2~4 combined with contralateral suppression and with adrenal nodule(s) on CT, while a diagnosis of NAT was made for LI < 2 or LI 2~4 without contralateral suppression and with adrenal nodule(s) on CT. A diagnosis of unilateral IHA required LI ≥ 4 or LI 2~4 combined with contralateral suppression, and with no nodule on CT. A diagnosis of bilateral IHA was made even if LI < 4, if the clinical diagnosis was PA, and with no nodule on CT 18.

### Synthesis of [^18^F]AlF-NOTA-T140 and ^18^F-AlF-NOTA-pentixather

[^18^F]AlF-NOTA-T140 or [^18^F]AlF-NOTA-pentixather was synthesized in a sterile hot cell. An AllinOne module (Trasis, Ans, Belgium) was used and modified. ^18^F was passed through a Sep-Pak light QMA cartridge and then eluted with 0.9% NaCl (0.5 mL). The eluate was added to a solution containing 25 μL of aqueous AlCl_3_ (2 mM in 0.5 M NaOAc, pH = 4), 100 μL of aqueous NOTA-pentixather (2 mg/mL), 300 μL of NaOAc buffer (0.5 M, pH = 4), and CH_3_CN (1 mL). The reaction mixture was heated at 100 °C for 15 min. After cooling to 40 °C, the mixture was diluted with deionized water, and the HLB was washed with 10 mL of deionized water to remove unreacted ^18^F and CH_3_CN. The final product was collected with 2 mL of 50% ethanol and passed through a sterile 0.22 mm filter with 10 mL saline. The radiochemical purity of [^18^F]AlF-NOTA-T140 or [^18^F]AlF-NOTA-pentixather was > 99%.

### [^18^F]AlF-NOTA-T140 PET/CT and [^18^F]AlF-NOTA-pentixather PET/CT scans

All images were obtained using a Siemens Biograph mCT Flow 64 PET/CT scanner. At 50-60 min after intravenous injection of [^18^F]AlF-NOTA-T140 (250.9 ± 35.92 MBq, specific activity 40 ± 25 GBq/μmol) or [^18^F]AlF-NOTA-pentixather (314.9 ± 57.4 MBq, specific activity 49 ± 29 GBq/μmol), non-contrast CT images were acquired from the top of the head to the middle thigh (120 kV, automatic mAs, slice width 3 mm, space 2 mm, screw pitch 0.8 mm). Then PET images from the top of the head to mid femur were acquired over 12 min using the flow method. Data were reconstructed using the TrueX + TOF (Gauss and Allpass filter) algorithm with a point-spread function correction using 2 iterations and 21 subsets, and a 256 × 256 matrix size. The images were smoothed by means of a 5 mm full width at half-maximum Gaussian filter. The section thickness of CT scans matched the PET slice thickness.

### Image analysis

PET/CT images were assessed by 2 experienced nuclear medicine physicians, who were blinded to the clinical information of the patients. A positive PET/CT lesion showed higher tracer uptake than normal adrenal tissue on CT, while a negative lesion showed equal or lower uptake. The PET images were analyzed visually and semi-quantitatively. The maximum standardized uptake value (SUVmax) of the adrenal lesions were measured by assigning spherical regions of interest (ROI) with a diameter of 1 cm. The lesion-to-liver ratio (LLR) on PET/CT was calculated by dividing the SUVmax of the lesion (ROI = 1 cm) by the mean standardized uptake value (SUVmean) of the normal liver (ROI = 2 cm), and the lesion-to-contralateral ratio (LCR) was calculated by dividing the SUVmax of lesion (ROI = 1 cm) by the SUVmean of the contralateral normal adrenal gland (ROI = 1 cm).

### Follow-up assessment

We collected postoperative data from patients who underwent surgery within 3 months. Serum aldosterone, serum potassium, and blood pressure levels were collected from the clinical database, and patient-reported symptom reports were obtained *via* telephone. According to the Primary Aldosteronism Surgical Outcome (PASO) study consensus 19, postoperative patients were divided into three groups: (1) cure: normal blood pressure < 140/90 mmHg without the aid of antihypertensive medication along with normal serum potassium and ARR; (2) improvement: unchanged high blood pressure with reduced antihypertensive medication, or lowered blood pressure with unchanged or reduced antihypertensive medication with normal serum potassium and ≥ 50% decrease in baseline plasma aldosterone concentration; and (3) no improvement: unchanged or increased blood pressure with the same or increased antihypertensive medication, persistent hypokalemia or raised ARR.

### Diagnosis of bilateral IHA without surgery

The diagnosis of bilateral IHA in non-surgical patients was based on the fulfillment of all of the following 5 criteria: (1) upright ARR > 30 (ng/dl)/(ng/ml/h), with or without hypokalemia; (2) post-saline infusion test plasma aldosterone concentration (PAC) > 10.0 ng/dL; (3) bilateral adrenal glands were normal or hyperplastic on thin-section CT; (4) A*VS.* indicating no aldosterone-secreting dominant side; (5) exclusion of renal artery stenosis and other factors that can cause elevated aldosterone, such as the use of large amounts of licorice.

### Immunohistochemistry

For postoperative immunohistochemical staining analysis of formalin-fixed paraffin-embedded tissue specimens, the two antibodies used and their dilutions were: CXCR4 (1:200, ab124824; Abcam) and CYP11B2 (1:200, ab168388; Abcam). Immunohistochemically stained sections with both antibodies were independently analyzed by experienced pathologists who were blinded to other clinical and examination information of the patients. Based on the percentage of positively stained cells, the staining results were categorized into 5 grades (score 0, no positive cells; score 1, ≤ 10% positive cells; score 2, 10-50% positive cells; score 3, 51-75% positive cells; and score 4, > 75% positive cells).

### Pathological diagnostic criteria

For comparison with [^18^F]AlF-NOTA-pentixather PET/CT results, we developed pathological diagnostic criteria for several adrenal diseases. For APA diagnosis, the criteria were postoperative pathology identifying adrenocortical adenoma and positive staining (score, 2-4) of CYP11B2 in adenoma cells. For IHA, the criteria were postoperative pathology identifying adrenocortical hyperplasia and no or weak CYP11B2 expression in hyperplastic cells (score, 0-1). For NAT, the criteria were postoperative pathology identifying adrenocortical adenoma or other types of tumors and no or weak CYP11B2 expression in tumor cells (score, 0-1).

### Statistical analysis

Descriptive statistics were used to determine the means, standard deviations (SD), and ranges. The Mann-Whitney U test was used to evaluate differences between groups. The sensitivity, and specificity of [^18^F]AlF-NOTA-pentixather PET/CT in detecting APA, IHA and NAT were also calculated. The Pearson correlation coefficient was used to assess the correlation between the proportion of CXCR4 positivity and SUVmax of adrenal lesions, the proportion of CXCR4 positivity and proportion of CYP11B2 positivity, and SUVmax and other characteristics of APA, AVS and [^18^F]AlF-NOTA-pentixather PET/CT results. The P value between four different follow-up groups was calculated *via* one-way analysis of variance and chi-square tests.

## Results

### Comparison of [^18^F]AlF-NOTA-T140 and [^18^F]AlF-NOTA-pentixather PET/CT

**Figure 1:**
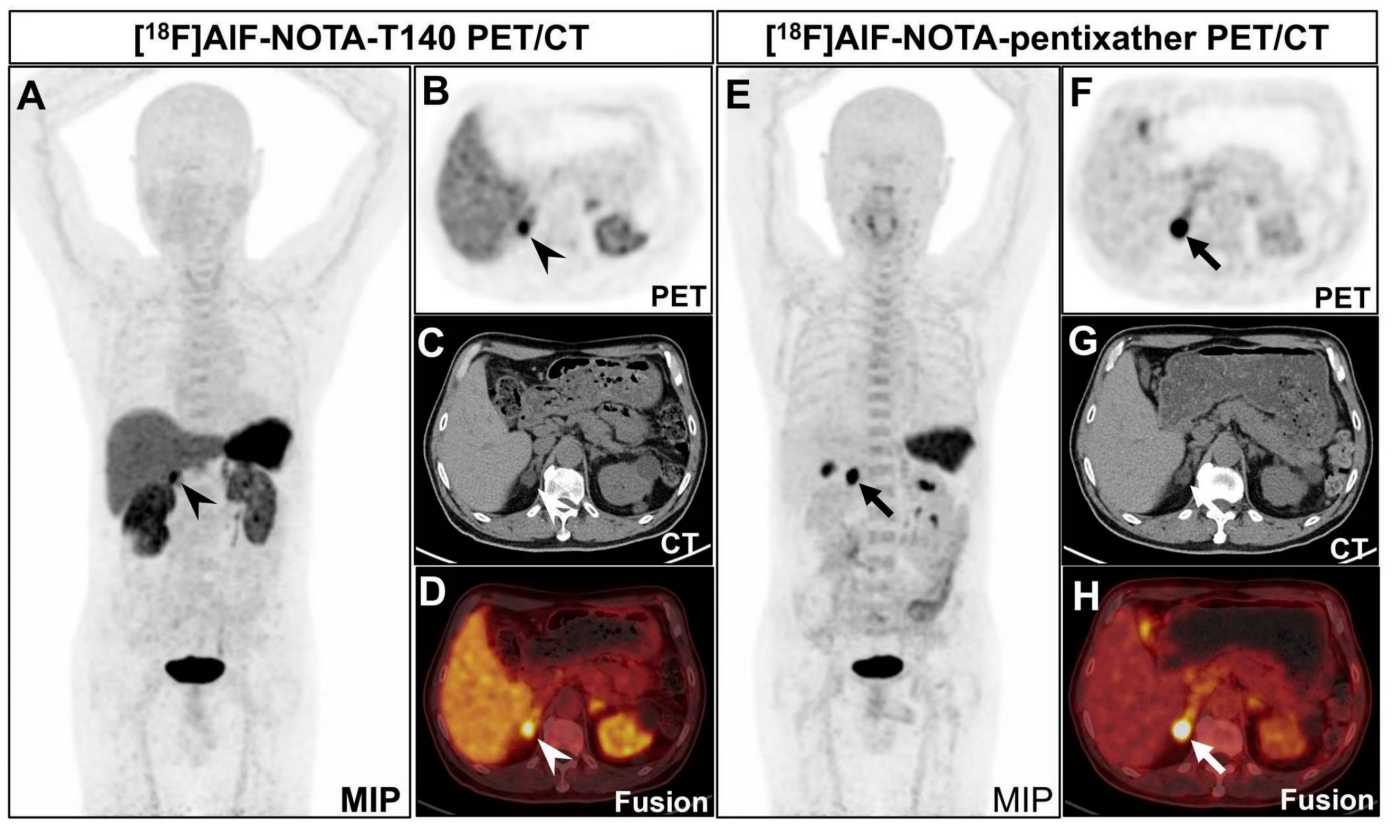
A 70-year-old man was diagnosed with PA. CT revealed a nodule in the right adrenal gland, measuring about 1.7×1.9 cm. He underwent both [^18^F]AlF-NOTA-T140 PET/CT and [^18^F]AlF-NOTA-pentixather PET/CT subsequently. **A-D:** [^18^F]AlF-NOTA-T140 PET/CT showed a SUVmax of 14.38 of the nodule (arrowheads). The background of liver (SUVmean 5.29) and kidney (right: SUVmean 7.23, left: SUVmean 6.15) was high. The patient's left ureter is narrow and the left kidney is atrophic, which may be why the left kidney took up less radionuclide than the right kidney. The patient has multiple cysts scattered in both kidneys, explaining the uneven distribution of radioactivity in both kidneys. The background of bone marrow was relatively low (SUVmax 3.07). **E-H:** [^18^F]AlF-NOTA-pentixather PET/CT showed a SUVmax of 26.90 of the nodule (thickheads). The background of liver (SUVmean 1.69) and kidney (SUVmean 2.63) was low. The background of bone marrow was relatively high (SUVmax 4.89). Postoperative pathology confirmed that the nodule is an adrenal adenoma.

Clinical trials have not yet been conducted to differentiate APA from non-APA lesions using [^18^F]AlF-NOTA-pentixather PET/CT or [^18^F]AlF-NOTA-T140 PET/CT. In our initial phase, we enrolled 3 PA patients and performed PET/CT imaging using [^18^F]AlF-NOTA-T140 and [^18^F]AlF-NOTA-pentixather in two days. The results showed significant statistical differences in SUVmax (mean 13.55 *vs.* 26.12) and LLR (mean 2.34 *vs.* 15.66) between [^18^F]AlF-NOTA-T140 PET/CT and [^18^F]AlF-NOTA-pentixather PET/CT (*P* < 0.05 and *P* < 0.001, respectively) (**Table 1** and **Figure 1**). However, there was no statistical difference in LCR (mean 2.26 *vs.* 5.43, *P* = 0.167). No patient in the study cohort experienced adverse events after injection of [^18^F]AlF-NOTA-pentixather or [^18^F]AlF-NOTA-T140.

### Patient cohort characteristics

**Figure 2:**
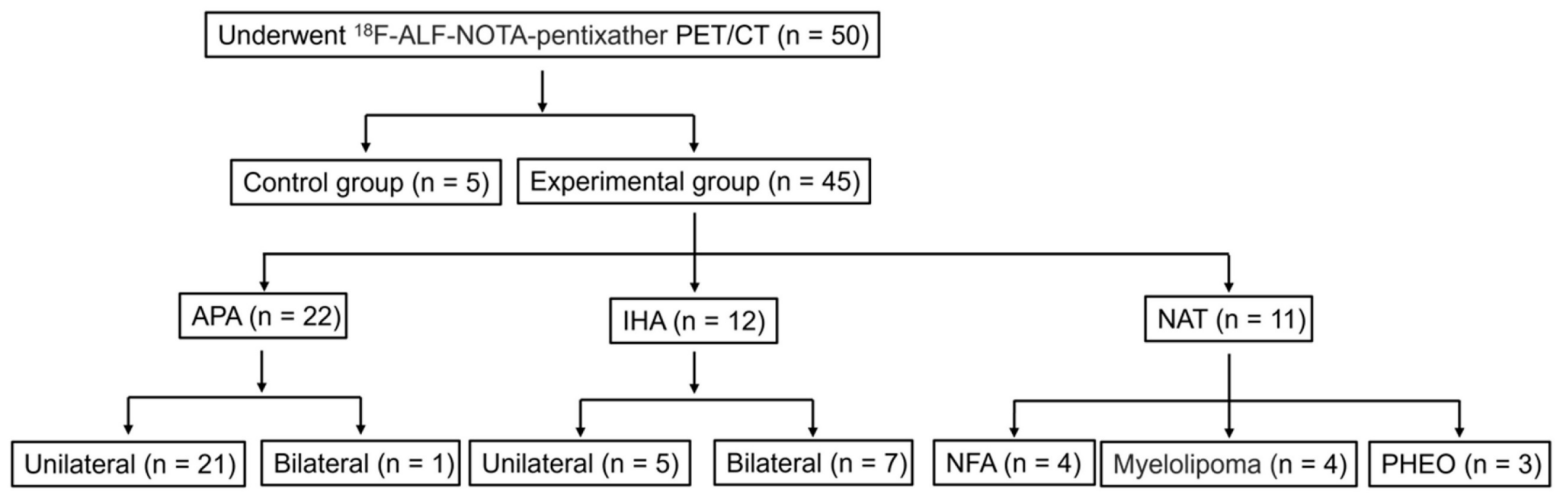
Patient stratification. *NAT (non-secreting aldosterone tumors, includes NFA, PHEO and myelolipoma).

To improve participant compliance and experimental feasibility, we exclusively enrolled PA patients to undergo [^18^F]AlF-NOTA-pentixather PET/CT. From January 2022 to October 2023, 50 patients were recruited for [^18^F]AlF-NOTA-pentixather PET-CT (**Figure 2**). The age at scanning ranged from 28 to 75 years (SD 51.20 ± 12.43 years). Among these subjects, 5 were suspected of having myeloma, but [^18^F]AlF-NOTA-pentixather PET-CT revealed no lesions. These subjects served as controls, in which the SUVmax of the right and left adrenal glands ranged from 6.57 to 7.13 (mean SUVmax: 6.78 ± 0.31) and 6.49 to 7.13 (mean SUVmax: 6.79 ± 0.32), respectively. The final analysis included a cohort of 45 patients with suspected PA (***Figure 2***). Among the 45 patients, 32 (71.1%) underwent AVS within 2 months prior to the PET/CT scan, with 15 showing unilateral dominance, 10 showing bilateral adrenal aldosterone secretion, and 7 experiencing right adrenal vein catheterization failure. 38 patients (84.4%) underwent surgery. Based on postoperative pathology and immunohistochemical staining results, the patients were grouped as follows: 22 with APA, 5 with IHA, 4 with NFA, and 7 with other tumors (**Figure 2**). Additionally, 7 IHA patients did not undergo surgery and were diagnosed based on clinical, radiological, and AVS results. Among the PA patients, 30 out of 34 cases (88.2%) had hypertension, with 23 cases (67.6%) having hypertension poorly responsive to medication and 10 out of these 23 cases (43.5%) having resistant hypertension. Two (50%) NFA patients also had hypertension. Three cases (100%) of PHEO patients had hypertension, and 3 cases (75.0%) of myelolipoma patients had hypertension. In addition, 24 out of 34 PA patients (70.6%) had hypokalemia. Furthermore, 29 out of 34 PA patients (85.3%) had positive captopril test results. The average length of the lesions determined by CT scan was 1.58 ± 0.85 (0.6-4.7) cm. Detailed baseline characteristics are shown in **Table 2**.

### The diagnostic efficacy of [^18^F]AlF-NOTA-pentixather PET/CT for APA

**Figure 3:**
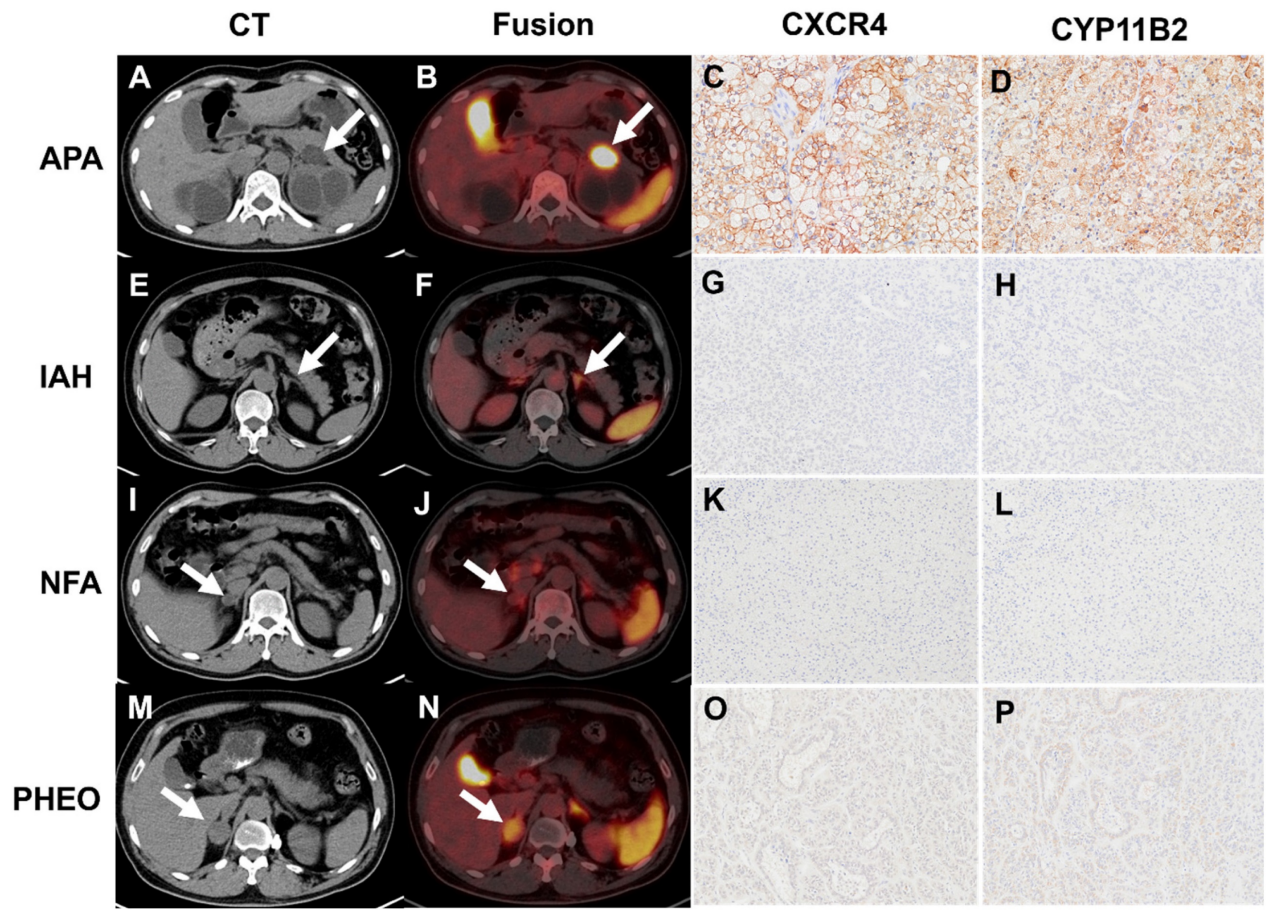
**A-D:** A true positive uptake (SUVmax 50.48, white arrow) was observed in one APA lesion with strong expression of CXCR4 and CYP11B2. **E-H:** [^18^F]AlF-NOTA-pentixather PET/CT was true negative in IHA (SUVmax 10.65, white arrow) with no elevated expression of CXCR4 and CYP11B2. **I-L:** Representative [^18^F]AlF-NOTA-pentixather PET/CT of true-negative NFA lesion (SUVmax 2.80, white arrow) with no elevated expression of CXCR4 and CYP11B2. **M-P:** [^18^F]AlF-NOTA-pentixather PET/CT of PHEO (SUVmax 11.30, white arrow) with a few expressions of CXCR4 and CYP11B2.

**Figure 4:**
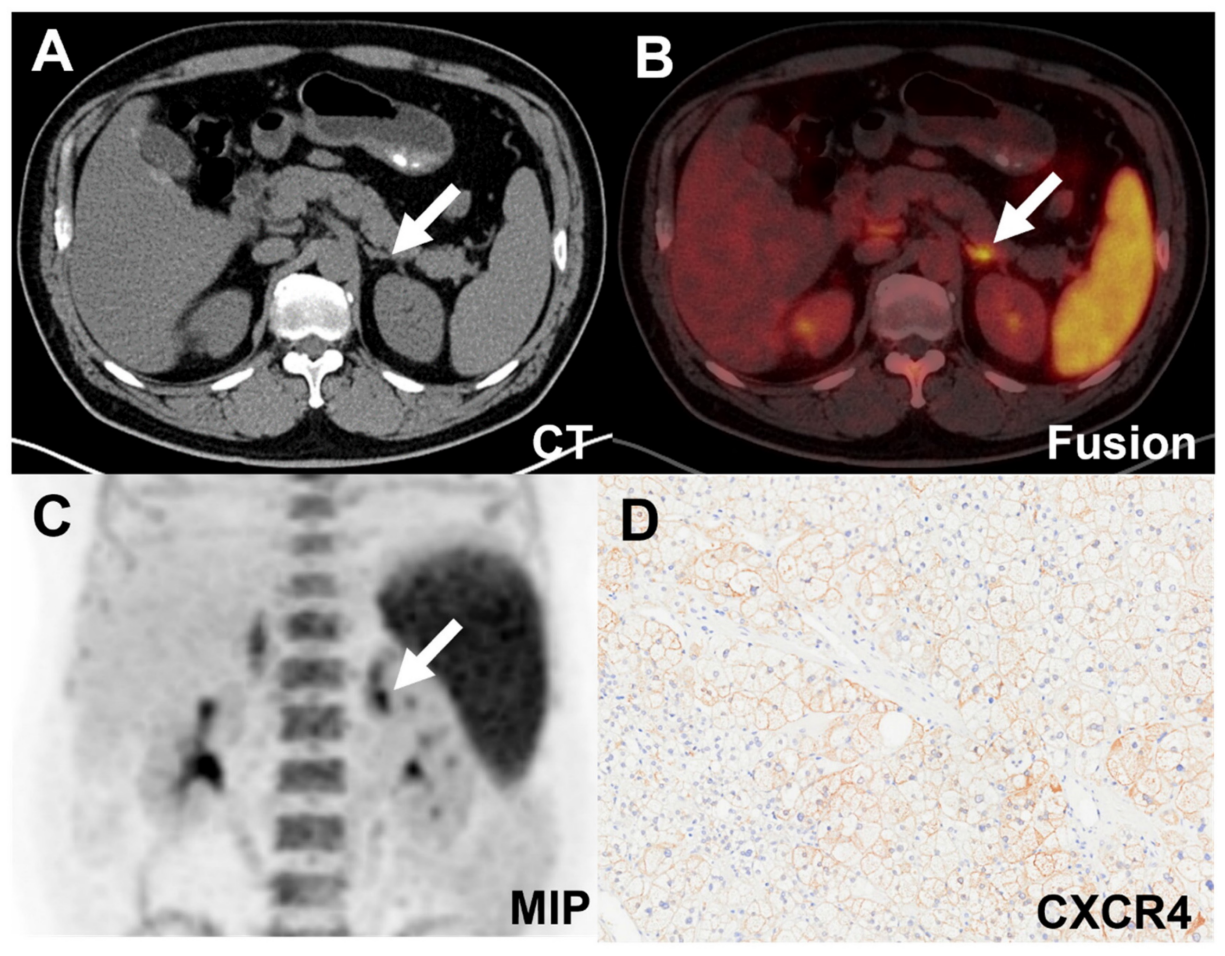
False-positive uptake (SUVmax 11.08, white arrow) in an IHA lesion on [^18^F]AlF-NOTA-pentixather PET/CT scan. **A-C.** PET/CT showed increased activity in the left adrenal nodule. **D.** Expression of CXCR4 was demonstrated in the adrenal hyperplastic tissue.

Forty-five patients were included, resulting in a total analysis of 48 lesions. [^18^F]AlF-NOTA-pentixather PET/CT showed visual positivity in 26 out of 48 lesions. All 24 APA lesions exhibited visual positivity (**Table 3** and **Figure 3**), while 2 IHA lesions showed a false-positive uptake (**Table 3** and** Figure 4**). Ten IHA lesions and 2 PHEO lesions showed [^18^F]AlF-NOTA-pentixather uptake comparable to normal adrenal glands (**Figure 3**). Three myelolipoma lesions exhibited no radiotracer uptake internally, with peripheral uptake similar to that of normal adrenal glands, subsequently confirming them as myelolipoma with concomitant adrenal hyperplasia. Additionally, 1 myelolipoma, 1 PHEO, and 5 NFA showed significantly lower uptake of the radiotracer compared to normal adrenal glands (**Figure 3**).

**Figure 5:**
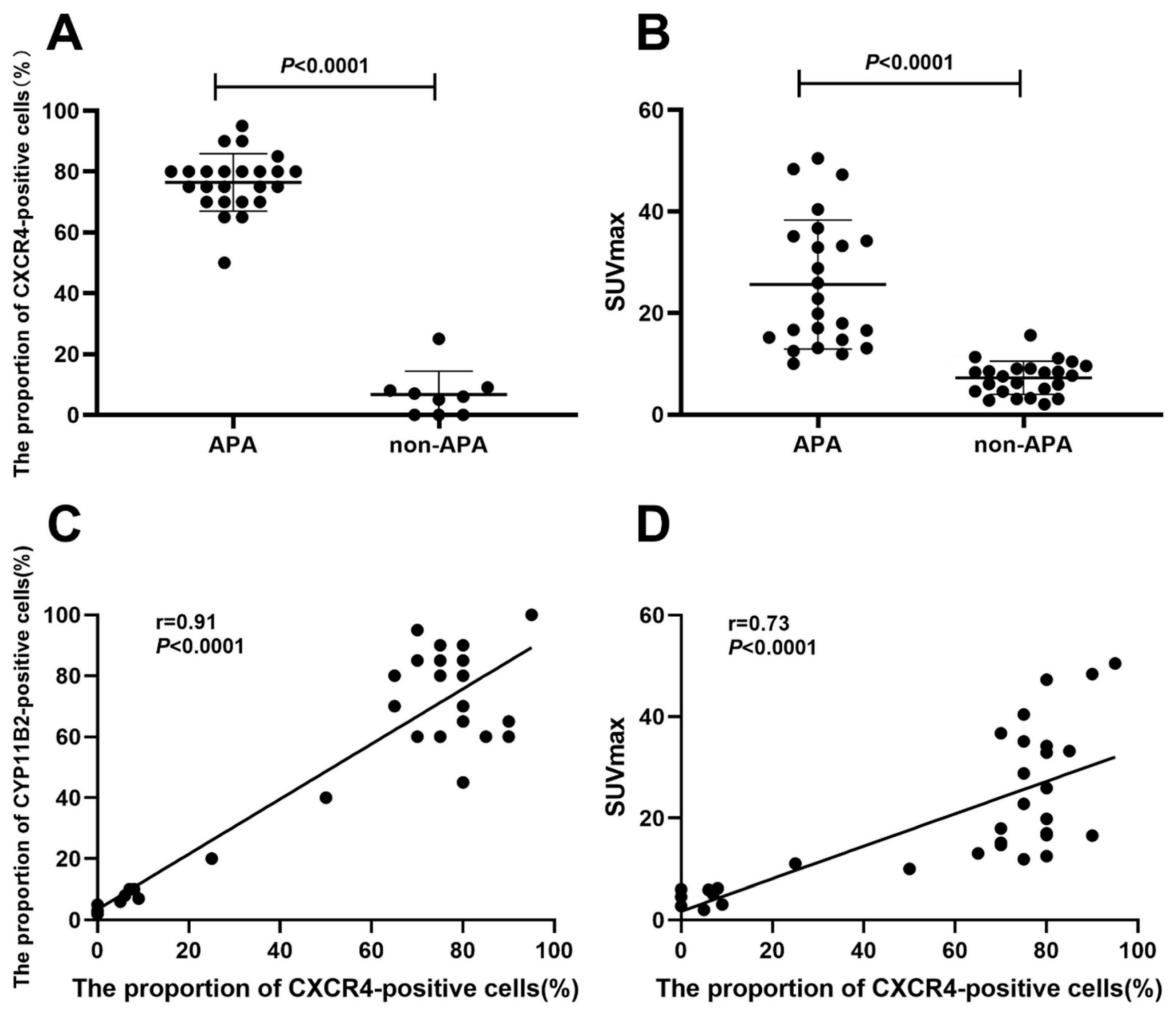
**A.** The proportion of CXCR4-positive cells (%) in APA lesions was significantly higher than that in non-APA lesions (*P* < 0.0001). **B.** The SUVmax of APA lesions (25.62 ± 12.71) was considerably higher than that of non-APA lesions (7.24 ± 3.27) (*P* < 0.0001). **C.** A significant relationship was seen between CXCR4 and CYP11B2 expression percentages (*r* = 0.91, *P* < 0.0001). **D.** A moderate correlation was seen between [^18^F]AlF-NOTA-pentixather PET SUVmax and CXCR4 expression (*r* = 0.73, *P* < 0.0001).

The sensitivity, specificity, and accuracy of [^18^F]AlF-NOTA-pentixather PET/CT in visually distinguishing APA from non-APA lesions were 100% (95% confidence interval [CI], 82.8-100), 91.7% (95% CI, 71.5-98.5), and 95.8%, respectively (**Table 3**). A high tracer uptake was detected in APA lesions on [^18^F]AlF-NOTA-pentixather PET/CT with an SUVmax of 25.62 ± 12.71 (n = 24), which was significantly higher than non-APA lesions (7.24 ± 3.27, n = 24) (*P* < 0.0001) (**Figure 5**). In non-APA lesions, the SUVmax was 9.02 ± 2.57 for the 12 IHA lesions and 5.60 ± 3.04 for the 12 NAT lesions. The optimal cutoff value of SUVmax was 11.60 calculated by ROC analysis, with a sensitivity of 95.8% (95% CI, 79.8-99.8) and specificity of 96.0% (95% CI, 80.5-99.8). The area under ROC curve (AUC) was 0.98 (95% CI, 0.96-1.00, *P* < 0.0001). The LCR and LLR of APA lesions were 4.92 ± 2.91 and 13.26 ± 7.15, respectively, while those of non-APA lesions were 1.02 ± 0.32 and 3.60 ± 1.49, respectively. There were significant differences in LCR and LLR between the two groups (*P* < 0.0001). The cutoff value for LCR was 1.38, with a sensitivity of 95.8% (95% CI, 79.8-99.8) and specificity of 92.0% (95% CI, 75.0-98.6), while the cutoff value for LLR was 5.28, with a sensitivity and specificity of 91.7% (95% CI, 74.1-98.5) and 92.0% (95% CI, 75.0-98.6), respectively. The AUCs for LCR and LLR were 0.97 (95% CI, 0.91-1.00) and 0.97 (95% CI, 0.91-1.00), respectively (**Table 3**).

### Correlation of [^18^F]AlF-NOTA-pentixather PET/CT and clinical characteristics with follow-up outcomes

We collected follow-up data from 34 PA patients and 7 symptomatic non-APA patients (6.5 ± 3.0 months) to evaluate the correlation between [^18^F]AlF-NOTA-pentixather PET/CT and clinical results, including outcomes, during the follow-up period. All PET-positive patients with lesions (n = 24) benefited from surgical treatment, with 21/24 (87.5%) patients experiencing a cure of their hypertension, while the remaining 3/24 (12.5%) patients experienced some improvement in their hypertension. Among the 17 patients with PET-negative lesions, 4/17 (23.5%) patients were cured, 7/17 (41.2%) patients improved after surgery, and 6/17 (35.3%) patients suffered persistent hypertension (**Table 4**). PET/CT visual analysis (positive uptake/no increased uptake) results highly correlated with hypertension follow-up assessments (cure/improvement/no improvement) (χ^2^ = 11.58, *P* = 0.003). The SUVmax of cured patients was higher than that of patients with improvement or no improvement (*P* = 0.007) (**Table 4**).

### The correlation of immunohistochemistry findings and SUVmax of [^18^F]AlF-NOTA-pentixather PET/CT

Using immunohistochemical methods, we detected the expression of CXCR4 and CYP11B2 in 24 cases of APA, 4 cases of IHA, and 5 cases of NAT lesion tissues. The proportion of CXCR4-positive cells in APA lesions (CXCR4-positive cells/total cells) was significantly higher than that in non-APA lesions (*P* < 0.0001) (**Figure 5A**). Furthermore, the expression of CXCR4 was significantly correlated with CYP11B2 (r = 0.91, *P* < 0.0001). In addition, we observed a moderate correlation between the SUVmax of [^18^F]AlF-NOTA-pentixather PET/CT and the proportion of CXCR4-positive cells (*r* = 0.73, *P* < 0.0001) (**Figure 5D**). The correlation coefficient between ARR values and the proportion of CXCR4-positive cells was 0.50 (*P* = 0.002).

### The correlation between APA SUVmax and clinical characteristics

The correlation analysis results of SUVmax in 24 APA lesions with clinical characteristics are shown in **Table 5**. The obtained correlation coefficients were: plasma aldosterone concentration *r* = 0.56 (*P* = 0.01), ARR *r* = 0.52 (*P* = 0.02), serum potassium *r* = 0.30 (*P* = 0.18). The correlation coefficients of other factors were all less than 0.3.

### The correlation between [^18^F]AlF-NOTA-pentixather PET/CT results and AVS results

A total of 25 patients (78.1%) successfully underwent AVS, with 15 individuals showing unilateral dominance (13 APA, 2 IHA) and 10 individuals showing no apparent dominance (1 APA, 7 IHA and 2 NFA). Seven patients (21.9%) experienced failure during right adrenal vein catheterization. The concordance rate between PET/CT positive results and AVS was 100%.

## Discussion

Fluorine-18 has advantages over gallium-68, including well-established centralized cyclotron production with theoretically unlimited radiotracer availability; its longer half-life, which allows for transport to more distant centers and the possibility of delayed imaging; and its higher positron yield and shorter positron range in tissues, which can result in improved image resolution 20. Therefore, in addition to the numerous studies on using ^68^Ga-labeled CXCR4 radiopharmaceuticals for APA diagnosis, it is still essential to explore the potential of ^18^F-labeled CXCR4.

Preliminary research suggests that hypomethylation of G protein-coupled receptor genes, resulting in enhanced gene expression and transcription, may be the reason for the autonomous overproduction of aldosterone in APA, potentially linking CXCR4 expression to aldosterone production 21. Our findings align with previously published studies on ^68^Ga-Pentixafor PET/CT 12, 13, 22, 23, which sought to clinically assess the efficacy of ^68^Ga-pentixafor PET/CT in identifying APA and conclude that ^68^Ga-Pentixafor PET/CT is a highly sensitive and specific non-invasive method for localizing and characterizing PA. Despite our inclusion of other tumors causing PA symptoms besides NFA in the analysis, the results of these tumors on PET/CT were all negative, which is consistent with the classification of results by NFA. Therefore, PHEO and myelolipoma cases did not impact the interpretation of positive results of [^18^F]AlF-NOTA-pentixather PET/CT.

The [^18^F]AlF-NOTA-pentixather PET/CT findings strongly align with postoperative results. Of the 24 PA patients with positive lesions who underwent adrenalectomy, 79.2% (19/24) achieved a cure. One IHA patient exhibited focal uptake in the left adrenal region, with an SUVmax of 11.08, and was cured post-unilateral adrenalectomy. Immunohistochemistry revealed aldosterone-producing cell clusters, likely contributing to hypertension and hypokalemia 24. Research suggests that nodular hyperplasia and aldosterone-producing cell clusters near adenomas blur the line between unilateral aldosterone-producing adenomas and bilateral adrenal hyperplasia 24, 25. PA patients show high nodule uptake and elevated adrenal background, possibly due to the presence of bilateral adrenal mild hyperplasia in addition to unilateral APA, which is also an advantage of metabolic imaging.

In PA diagnosis, the ARR is commonly employed for its non-invasive nature and ease of repeated testing, yet its reliability is questionable due to low reproducibility and wide sensitivity (10-100%) and specificity (70-100%) ranges, compounded by a lack of standardization in testing methods 26. In our study, an ARR ≥ 30 (ng/dl)/(ng/ml/h) diagnosed PA with a sensitivity of 97.3% and a specificity of 33.3%. One patient, initially suspected of APA, showed no increased adrenal uptake on PET/CT and was ultimately classified as NFA based on histology, CYP11B2 immunostaining, and subsequent evaluations.

There was a significant correlation between SUVmax and plasma aldosterone or ARR and there was no correlation between SUVmax and age, duration of hypertension, systolic pressure, diastolic pressure, duration of hypokalemia, serum potassium, or tumor length on CT, suggesting that [^18^F]AlF-NOTA-pentixather PET/CT has good reproducibility. This also indicates that SUVmax cannot be entirely predicted based on tumor size, evidenced by smaller tumors presenting higher SUVmax than some larger ones.

Traditional imaging methods like CT and MRI are commonly used to detect PA lesions, but their accuracy is limited, particularly in distinguishing between APA and NFA 27. This differentiation is crucial for guiding treatment decisions and avoiding unnecessary surgeries 28. CT, for example, has a detection accuracy of only 64.4%, and it typically fails to detect tumors smaller than 1 cm in diameter 29, 30. In contrast, [^18^F]AlF-NOTA-pentixather PET/CT provides better sensitivity and specificity by capturing molecular details of CXCR4 and PA lesions. This imaging modality is particularly beneficial for cases where CT struggles, especially in patients with unclear adrenal lesions. There were 6 APA patients with adrenal nodules smaller than 1 cm, [^18^F]AlF-NOTA-pentixather PET/CT demonstrated an average SUVmax of 22.5 ± 7.5, average LCR of 3.5 ± 1.6 cm, and average LLR of 12.7 ± 5.5, making these nodules highly visible on PET scans.

AVS is the gold standard for APA diagnosis, yet it is subject to many variables, has mixed success rates, and is invasive 7. In our group, 32 patients had AVS, with a 21.9% failure rate (n = 7) due to right-side cannulation issues. These patients turned to [^18^F]AlF-NOTA-pentixather PET/CT, which successfully detected 5 APAs, later confirmed by postoperative pathology and cured surgically. Moreover, 2 patients with renal insufficiency, unable to have AVS, also underwent [^18^F]AlF-NOTA-pentixather PET/CT, and no side effects occurred. In our research, the concordance rate between [^18^F]AlF-NOTA-pentixather PET/CT and AVS in APA diagnosis was 100%, indicating the potential of [^18^F]AlF-NOTA-pentixather PET/CT as a non-invasive alternative to AVS.

Our preliminary small-scale study revealed significant statistical disparities in SUVmax of lesions and LLR between [^18^F]AlF-NOTA-T140 and [^18^F]AlF-NOTA-pentixather at the 60-minute post-injection, favoring the latter. However, the bone marrow uptake of [^18^F]AlF-NOTA-T140 was notably lower compared to [^18^F]AlF-NOTA-pentixather (**Figure 1**). This finding indicated that when radiolabeled with a therapeutic radionuclide, T140 may hold therapeutic advantages over those of pentixather. Additionally, the high liver and spleen background observed at the 60-minute time point for [^18^F]AlF-NOTA-T140 can be attributed to its prolonged biological half-life. Consequently, further multi-time-point imaging studies are warranted to determine the most effective imaging time point for [^18^F]AlF-NOTA-T140.

This unicentric prospective study has some limitations. Firstly, the limited number and unbalanced patient cohort in our study might lead to biased results. Secondly, most IHA patients received a composite diagnosis without histopathological confirmation, which may affect patient grouping and statistical accuracy. Thirdly, direct comparison with ^68^Ga-pentixafor PET/CT was not conducted.

## Conclusion

In this prospective study, the SUVmax in [^18^F]AlF-NOTA-pentixather PET/CT is closely associated with the molecular expression of CXCR4 and CYP11B2. The results of [^18^F]AlF-NOTA-pentixather PET/CT and AVS are highly consistent. Our findings indicated that [^18^F]AlF-NOTA-pentixather PET/CT is an effective and practical method for diagnosing APA lesions, demonstrating high sensitivity and accuracy.

## Figures and Tables

**Table 1:** The comparison of [^18^F]AlF-NOTA-T140 and [^18^F]AlF-NOTA-pentixather PET/CT

Parameters	[^18^F]AlF-NOTA-T140	[^18^F]AlF-NOTA-pentixather	*P* value
Mean SUVmax (n = 3)	13.55	26.12	0.007
Mean LLR (n = 3)	2.34	15.66	< 0.001
Mean LCR (n = 3)	2.26	5.43	0.167

**Table 2:** Baseline characteristics and therapy management of patients

Characteristics (median, range)	Total patients (n = 45)	APA patients (n = 22)	IHA patients (n = 12)	NAT patients (n = 11)
Age	50.6 ± 11.6 (28-70)	50.1 ± 12.8 (29-70)	46.8 ± 11.9 (28-69)	55.9 ± 6.5 (45-65)
Gender (male)	26	15	6	5
Number with hypertension	38	20	10	8
Number with poorly controlled hypertension	27	18	5	4
Duration of hypertension (year)	6.0 ± 5.9 (0.3-20)	7.5 ± 6.6 (0.3-20)	5.4 ± 5.6 (1.0-20)	3.3 ± 3.0 (0.5-10)
Systolic pressure (mmHg)	142.9 ± 19.5 (108-189)	148.3 ± 18.0 (121-189)	143.5 ± 20.0 (108-186)	128.8 ± 16.1 (111-155)
Diastolic pressure (mmHg)	90.1 ± 13.2 (65-110)	93.8 ± 12.4(69-110)	89.3 ± 14.4 (68-109)	82.1 ± 11.1 (65-96)
Number with hypokalemia	24	18	6	0
Duration of hypokalemia (years)	2.0 ± 1.6 (0.1-5.0)	2.3 ± 1.7 (0.1-5.0)	1.2 ± 1.1 (0.2-3.0)	/
Serum potassium (mmol/l)	3.4 ± 0.6 (2.0-4.8)	3.1 ± 0.5 (2.0-4.1)	3.6 ± 0.6 (2.8-4.8)	4.0 ± 0.3 (3.6-4.5)
Plasma aldosterone concentration (ng/dl)	30.9 ± 20.0 (7.4-9.2)	39.1 ± 23.5 (7.4-9.2)	24.5 ± 11.5 (10.4-48.9)	19.0 ± 8.4 (12.2-36.6)
ARR ([ng/dl]/[ng/ml/h])	124.9 ± 84.1 (19.8-351.2)	183.9 ± 81.1 (69.5-351.2)	78.5 ± 18.7 (53.3-113.2)	49.2 ± 22.4 (19.8-83.7)
Number with positive captopril tests	31	19/20	10/11	2/6
Number of successful AVS	25	14	9	2
Lesion location (right)	16	7	2	7
Lesion location (left)	21	14	3	4
Lesion location (bilateral)	8	1	7	0
Tumor length on CT (cm)	1.58 ± 0.85 (0.6-4.7)	1.54 ± 0.69 (0.6-3.6)	0.8 ± 0.2 (0.6-1.0)	1.96 ± 1.19 (1.0-4.7)

**Table 3:** [^18^F]AlF-NOTA-pentixather PET-CT for the diagnosis of aldosterone-producing adenomas

[^18^F]AlF-NOTA-pentixather PET-CT		APA lesions (n = 24)	Non-APA lesions (n = 24)
Visual analysis	Positive Lesions (n = 26)	24 (100%)	2 (8.3%)
	Negative Lesions (n = 22)	0 (0%)	22 (91.7%)
Semi-quantitative analysis			
SUVmax	≥ 11.60 (n = 23)	22 (91.7%)	1 (4.2%)
	< 11.60 (n = 25)	2 (8.3%)	23 (95.8%)
LCR	≥ 1.38 (n = 25)	23 (95.8%)	2 (8.3%)
	< 1.38 (n = 23)	1 (4.2%)	22 (91.7%)
LLR	≥ 5.28 (n = 24)	22 (91.7%)	2 (8.3%)
	< 5.28 (n = 24)	2 (8.3%)	22 (91.7%)

*Non-APA lesions (include IHA, NFA, PHEO, and myelolipoma.)

**Table 4:** The comparison of follow-up outcome with [^18^F]AlF-NOTA-pentixather PET/CT and final diagnosis (n = 41)

	Cure (n = 25)	Improvement (n = 10)	No Improvement (n = 6)	*P* value
[^18^F]AlF-NOTA-pentixather PET/CT				
Positive patients (n = 24)	21	3	0	< 0.05
Negative patients (n = 17)	4	7	6	
SUVmax	23.4 ± 14.1	12.7 ± 3.5	7.4 ± 1.9	< 0.05
Final diagnosis				
APA (n = 22)	19	3	0	< 0.05
IHA (n = 12)	2	5	5	
NAT (n = 7)	4	2	1	

*P < 0.05: statistical significance of the difference in outcome between the three groups (cure, improvement, and no improvement)

**Table 5:** Correlation between APA SUVmax and clinical characteristics

Clinical characters	Correlation Coefficient	*P* value
Age	-0.04	0.86
Duration of hypertension	-0.09	0.70
Systolic pressure	-0.18	0.46
Diastolic pressure	0.28	0.21
Duration of hypokalemia	0.12	0.23
Serum potassium	0.30	0.18
Plasma aldosterone concentration	0.56	0.01
ARR	0.52	0.02
Tumor length on CT	0.04	0.86

## Abbreviations

APA: aldosterone-producing adenomas
IHA: idiopathic hyperaldosteronism
NFA: nonfunctional adrenal adenoma
CXCR: chemokine receptor
PHEO: pheochromocytoma
PA: primary aldosteronism
AVS: adrenal venous sampling
SUVmax: maximum standardized uptake value
LLR: lesion-to-liver ratio
LCR: lesion-to-contralateral ratio
NAT: non-secreting aldosterone tumors
MTO: metomidate
CYP11B1: 11-β-hydroxylase
CYP11B2: aldosterone synthase
SDF-1: stromal cell-derived factor-1
ARR: aldosterone-to-renin ratio
IRB: Institutional Review Board
ACTH: adrenocorticotropic hormone
SI: selectivity index
LI: lateralization index
ACR: aldosterone-to-cortisol ratio
ROI: regions of interest
SUVmean: mean standardized uptake value
PASO: Primary Aldosteronism Surgical Outcome
PAC: plasma aldosterone concentration
SD: standard deviations
CI: confidence interval
AUC: area under ROC curve
